# Increased incidence of childhood type 1 diabetes during the COVID‐19 pandemic. Figures from an Italian tertiary care center

**DOI:** 10.1111/1753-0407.13298

**Published:** 2022-08-02

**Authors:** Riccardo Schiaffini, Annalisa Deodati, Novella Rapini, Valentina Pampanini, Stefano Cianfarani

**Affiliations:** ^1^ Diabetes Unit ‐ Dipartimento Pediatrico Universitario Ospedaliero Bambino Gesù Children's Hospital, IRCCS Rome Italy; ^2^ Dipartimento Medicina dei Sistemi University of Rome Tor Vergata Rome Italy; ^3^ Department of Women's and Children's Health Karolinska Institutet Stockholm Sweden

## Abstract

**Highlights**

- In 2021 as a whole we report an increase (22% to 35%) in incidence of type 1 diabetes compared to the years 2017 to 2020.
- A peak of incidence in the last 4 months of 2021, when the “fourth wave” of COVID‐19 peaked in Italy, has been observed.
- In the last quarter of 2021 there was a significant increased type 1 diabetes incidence in the age group under 12 years.
- We speculate that SARS‐CoV‐2 infection may have had an impact on type 1 diabetes incidence in children, especially in age groups not protected by vaccination.

Recently, the Centers for Disease Control and Prevention reported an increased risk of newly diagnosed diabetes over 30 days after SARS‐CoV‐2 infection among persons aged <18 years.^1^

As confirmed by the authors, the mechanism through which SARS‐CoV‐2 infection might facilitate the increase of diabetes is complex and, at least in type 1 diabetes, different pathogenetic processes could be involved, such as direct damage to the pancreatic beta cells or immune‐mediated damage, or a cascade of events in which the viral infection acts as a trigger.

Similarly, the results from the DPV (Diabetes Patienten Verlaufsdokumentation) Registry^2^ demonstrated a significant increase in the incidence of pediatric type 1 diabetes during the COVID‐19 pandemic. The authors compared two cohorts of newly diagnosed type 1 diabetic children and observed an increase in the observed incidence of type 1 diabetes as compared with the expected one in 2020 to the first 6 months of 2021. This increased incidence was significant in children aged <11 years.

Future studies will clarify if this relationship has a biological significance, if there is a clear pathogenetic mechanism, and if prevention strategies, including vaccination, for eligible persons in this age group are applicable.

With this letter we want to highlights the experience of the Diabetes Unit in “Bambino Gesù” Children's Hospital, Rome, one of the largest tertiary referral centers for type 1 diabetes in Italy, during the COVID‐19 pandemic.

In 2021 as a whole we report an increase in incidence of type 1 diabetes compared to the years 2017 to 2020: in particular the newly diagnosed cases from 2017 to 2021 were 89 (2017), 102 (2018), 99 (2019), 99 (2020), and 121 (2021) with an increase in 2021 compared to 2017 and 2018–2020 respectively of 35% and 22%.

We observed the peak of incidence in the last 4 months of 2021, when the “fourth wave” of COVID‐19 peaked in Italy. Between September and December 2021 we recorded 50 new diagnoses of diabetes, which represent 41% of all new diagnoses of the year.

Stratification of the newly diagnosed population by age groups showed that in the last quarter of 2021 there was a significant increased type 1 diabetes incidence in the age group under 12 years: in particular we observed that 88% of the newly diagnosed children were younger than 12 (44 patients out of 50) (Figure 1), which is significantly different from what we observed during the first 8 months of the year (43 patients out of 71, equal to 60% of the sample). None of the patients reported previous symptoms of COVID‐19 or contact with infected people.

**FIGURE 1:**
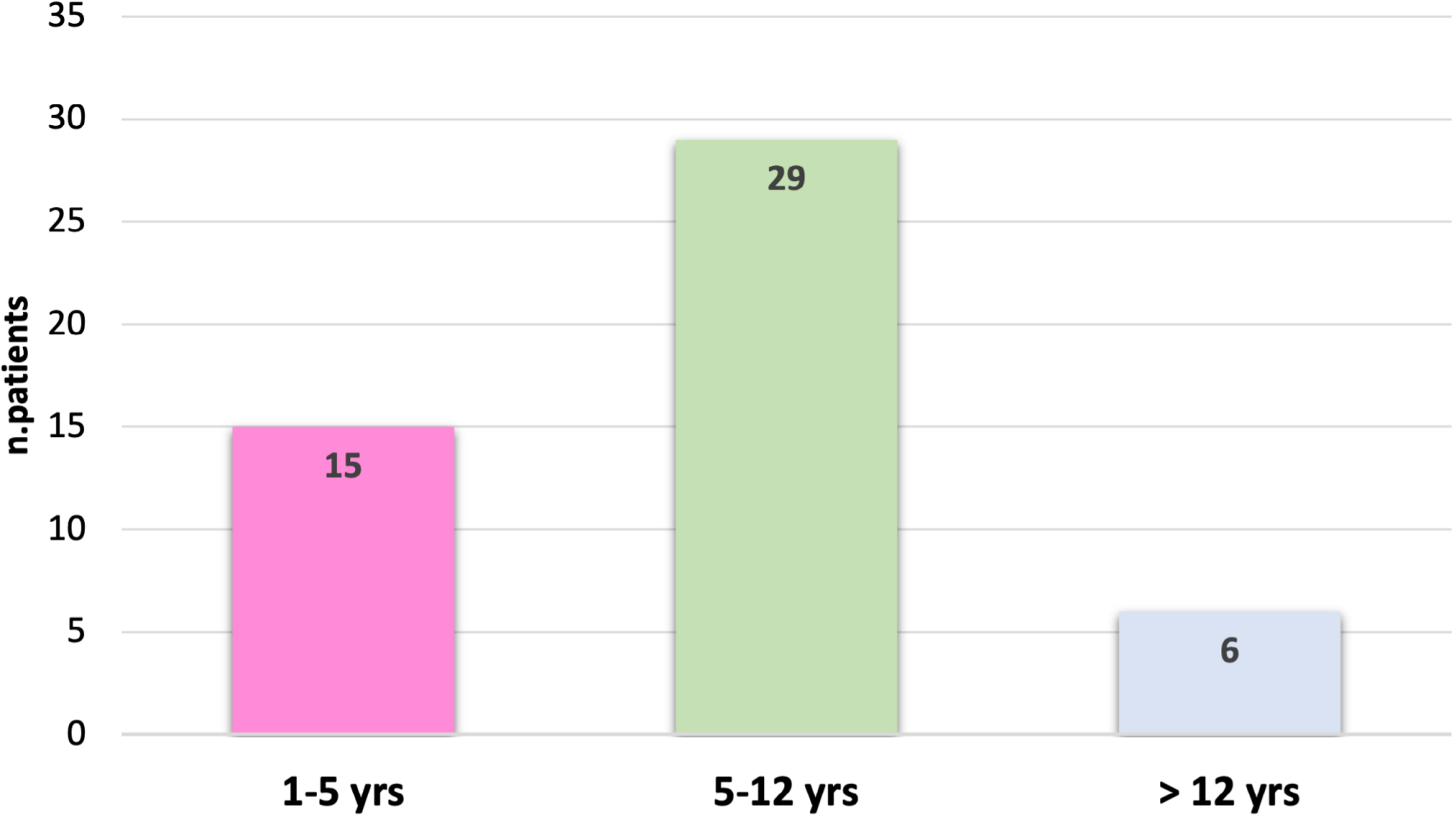
Number of patients with type 1 diabetes at onset during the last 2021 quarter, represented for age range; 1–5 years: age between 1 and 5 years; 5–12 years: age between 5 and 12 years; >12 years: age >12 years.

Finally, 25 newly diagnosed subjects in the last quarter of 2021 underwent serological tests for the presence of anti‐SARS‐CoV‐2 antibodies and 8 patients (32% of tested cases) were positive, suggesting a previous asymptomatic SARS‐CoV‐2 infection and a hypothetical involvement of SARS‐CoV‐2 infection in the pathogenesis of at least some cases of type 1 diabetes.

Pancreatic beta‐cell autoimmunity tested positive in almost all patients, with a frequency similar to all previous years considered for the analysis. In our laboratory at the onset of diabetes we regularly test the following four anti‐beta cell autoantibodies: GADA, IA2, ZNT8, and anti‐insulin. In particular in 2021 we found at least one positive beta‐cell autoantibody in 116 out of 121 patients, equal to 95.8% of our series. These data are comparable to those observed in other years.

In conclusion, we speculate that SARS‐CoV‐2 infection may have had an impact on type 1 diabetes incidence in children, especially in age groups not protected by vaccination.

Prospective and targeted population studies are needed to consolidate this hypothesis and to verify if vaccination for SARS‐CoV‐2 can be considered protective in the pathogenesis of childhood type 1 diabetes.
